# Polytobacco use among a nationally-representative sample of black high school students

**DOI:** 10.1186/s12889-021-10228-7

**Published:** 2021-01-23

**Authors:** Tamika D. Gilreath, Derek T. Dangerfield, Francisco A. Montiel Ishino, Ashley V. Hill, Renee M. Johnson

**Affiliations:** 1 Department of Health & Kinesiology, College Station, Texas A & M University, 267 Gilchrist, 4243 TAMU, College station, TX 77843-4243 USA; 2 Johns Hopkins School of Nursing, Baltimore, MD USA; 3 Division of Intramural Research, National Institute for Minority Health Disparities, Bethesda, MD 20892 USA; 4 Division of Adolescent and Young Adult Medicine, UPMC Children’s Hospital of Pittsburgh, Department of Pediatrics, University of Pittsburgh School of Medicine, Pittsburgh, PA USA; 5 Department of Mental Health, Johns Hopkins Bloomberg School of Public Health, Baltimore, MD USA

## Abstract

**Background:**

Studies of the patterns of polytobacco use have increased. However, understanding the patterns of using multiple tobacco products among Black adolescents is minimal. This study identified the patterns of polytobacco use among U.S. Black adolescents.

**Methods:**

Latent class analysis (LCA) was used to identify patterns of adolescent polytobacco use among a representative sample of Black youth from the 2017 Youth Risk Behavior Survey (*n* = 2782). Ever and recent (past 30 day) use of cigarettes, electronic cigarettes, cigars, and dip or chewing tobacco were used as latent class indicators. Multinomial regression was conducted to identify the association if smoking adjusting for sex, age, grade, and marijuana use.

**Results:**

Most students were in the 9th grade (29%), e-cigarette users (21%) and were current marijuana users (25%). Three profiles of tobacco use were identified: Class 1: Non-smokers (81%), Class 2: E-cigarette Users (14%), and Class 3: Polytobacco Users (5%). Black adolescent Polytobacco users were the smallest class, but had the highest conditional probabilities of recent cigarette use, e-cigarette use, ever smoking cigars or chewing tobacco. Ever and current use of marijuana were associated with increased odds of being in the e-cigarette user versus non-smoker group, and current marijuana use was associated with increased odds of polytobacco use (aOR = 24.61, CI = 6.95–87.11).

**Conclusions:**

Findings suggests the need for targeted interventions for reducing tobacco use and examining the unique effects of polytobacco use on Black adolescents. Findings confirm a significant association of marijuana use with tobacco use.

## Background

Tobacco use is the single largest contributor to preventable deaths in the United States and is responsible for a considerable portion of excess morbidity and mortality among Black Americans [1–6]. Racial/ethnic differences in smoking-related health problems stem from a variety of factors, including smoking prevalence, types of products smoked (e.g., menthol cigarettes), differences in the nicotine metabolism rate, and social adversity that makes quitting more difficult for some racial/ethnic groups such as Black Americans [7–10]. The addictive characteristics and health effects of tobacco use are more difficult to treat with longer and greater exposure [11], thus Blacks who used tobacco in adolescence are at higher risk for increased adverse health outcomes across their life-course that contribute to health disparities in the United States.

### Tobacco use among U.S. high school students

Recent (past 30-day) use of any tobacco product among U.S. high school students declined from 24.2% to 19.6.% between 2011 to 2017 [12]. However, data from 2019 indicate that this has increased to 23% [13] Black youth had the lowest prevalence of any current tobacco product use (25.4%) compared to White (35.6%) and Latinx (26.6%) high school students in 2019 [13]. Driving the increase in recent tobacco product use is The use of electronic cigarettes. E-cigarette use among this population has increased drastically since their introduction in 2007 [14] and it has been estimated that 27.5% of high school students report recent use of e-cigarettes [13]. Further, 10.8% of high school students concurrently use more than two tobacco products [13]. Black youth have the second highest prevalence of polytobacco use (11.5%) compared to White youth (12%) and Latinx youth (8.5%) [13]. Given the evolving mix of tobacco/nicotine products available to youth, research that investigates polytobacco use is needed. Because adolescence is a crucial period for tobacco use initiation, characterizing the unique patterns of tobacco use among Black youth is an important step to developing prevention programs for the modern tobacco era.

### Co-use of marijuana and tobacco and black youth

Epidemiological studies have consistently found an association between tobacco and marijuana use in youth and young adults [15, 16]; however, Black youth are more likely to report co-use [15, 17, 18]. Co-use of tobacco and marijuana is of particular interest given the documented disparities in tobacco-related health outcomes [1, 11] as well as in health and social outcomes of marijuana use [19] experienced by U.S. Blacks. Further, in the context of the changing landscape of both tobacco and marijuana legislation, examining the likelihood of polytobacco use (use of more than two tobacco products) is important.

Given the evolving landscape, more detailed examination of the patterns of polytobacco use is important in order to assess the health effects associated with different combinations of use [20–22]. Despite the importance of reducing tobacco health disparities, the literature examining current polytobacco use exclusively among Black adolescents is limited [23, 24]. As a burgeoning area, it has become clear that some youth and adults are using multiple products concurrently. In addition to long-term health issues, it is also important to recognize that tobacco use may be a marker of risk for proximal behavioral health problems in adolescence, such as poor academic achievement, depressive symptoms, and lack of social integration among adolescent Blacks [11]. Additionally, Black adolescents who initiate smoking are more likely to leave home at a younger age and have less familial interaction transitioning into adulthood [25].

### The current study

Although studies on the patterns of polytobacco use have increased, most of these studies used cumbersome combination models that limited the ability to characterize patterns of co-occurring use [26–28]. Our purpose is to determine the patterns of polytobacco use among U.S. Black adolescents. Latent class analysis (LCA) is useful in identifying homogeneous subgroups within a heterogeneous population regarding the manifestations of a set of characteristics, such as the likelihood of using multiple tobacco products [29]. Given the known associations of tobacco and marijuana use particularly among U.S. Black youth, we examined marijuana use as it relates to profiles of tobacco use behaviors. We also examined differences in profiles of tobacco use by sex and grade level because use has been historically higher among boys and older adolescents. This study fills an important gap in the literature about patterns of tobacco use among Black youth for the e-cigarette era. Because Blacks experience disproportionate health problems from their tobacco use, our findings will provide epidemiological data that can be used to inform policy development and prevention programs.

## Methods

Data from this study come from the 2017 U.S. Centers for Disease Control and Prevention (CDC) Youth Risk Behavior Surveillance System (YRBS), a biennial survey to monitor health behaviors including substance use, violence, sexual risk behaviors, and mental health among 9th–12th graders. The survey utilizes a three-stage cluster sampling design to yield representative estimates of the prevalence of risk behaviors among high school students [30]. Our study used the 2017 survey and included students who self-identified as non-Hispanic Black (i.e., multi-racial youth were not included), and excluded respondents missing data on key variables (*n* = 2782) from the total sample of 14,765. The analysis of this publically available non-identifiable data was not subject to review by institutional review board pursuant to United States Code of Federal Regulations §46.101 and §46.104.

### Latent class indicators

The 2017 YRBS included 95 items covering a range of domains including substance use, mental health and other health-related behaviors. Tobacco use was measured by the following items:

#### Cigarette use

Participants were asked “Have you ever tried smoking, even one or two puffs?” Those who indicated cigarette use were asked to identify the number of days of cigarette use in the last 30 days. Responses were recoded to indicate “Never,” “Ever,” and “Recent (past 30 days)” use.

#### E-cigarette use

Participants were asked to identify whether they had ever used any electronic vapor products. Then, among those who indicated e-cigarette use, youths were asked to identify the number of days within the past 30 days they used e-cigarette products. Responses were recoded to indicate “Never,” “Ever,” and “Recent (past 30 days)” use.

#### Cigar use

Participants were asked on how many days in the past 30 days they had smoked cigars, cigarillos, or little cigars. Responses were dichotomized to indicate “No,” or “Yes” use.

#### Dip/chewing tobacco use

Participants indicated how many days they used chewing tobacco, snuff, dip, snus, or dissolvable tobacco products in the past 30 days. Responses were dichotomized to indicate “No,” or “Yes” to indicate use in the past 30 days.

### Covariates for latent class membership

Variables used to examine correlates of membership in latent classes included sex, grade (9th through 12th) and marijuana use. Sex was determined by asking respondents “What is your sex?” Response options were limited to “Male” or “Female.” Students indicated what grade in school they were in. Students were asked two questions related to marijuana use, which were combined and recoded to indicate “Never,” “Ever,” or “Recent” marijuana use.

### Statistical analysis

LCA was utilized to explore and identify tobacco use profiles among Black adolescents using cigarette smoking, e-cigarette use, other combustible tobacco products, and other non-combustible tobacco products as latent class indicators. A series of latent class models specifying one to five classes was tested. Optimal model selection was based upon recommended indices including low Adjusted Bayesian Information Criterion (aBIC) relative to other models, significant Lo-Mendell-Rubin Likelihood Ratio Test (LMR/LRT), and acceptable quality of classification [31]. aBIC is based on the loglikelihood of each model with the lowest value providing support to select a particular model. The LMR/LRT tests for improvement of fit for the model under consideration compared with a model with one less class. A *p*-value greater than 0.05 indicate that the model with one less class fits best. All analyses were conducted using Mplus Version 8.1 [32]. The Mplus tools stratification, cluster, and weight were used to calculate the correct standard errors for the complex survey design of the YRBS; data were weighted to represent the U.S. population. Missing data for latent class indicators were accounted for using the full information maximum likelihood (FIML) capabilities of Mplus. After determining the appropriate number of classes, multinomial logistic regression was used to assess the role of sex, grade level, and marijuana use in association with tobacco use class membership [33]. Covariates were treated as auxiliary variables using the R3STEP function in Mplus, which initiates the multinomial regression and maintains the class structure while controlling for uncertainty in class assignment [33]. Post hoc cross tabulations to explore tobacco product use within class were conducted in SAS 9.4 and represent unweighted data.

## Results

As shown in Table 1, the sample was balanced by sex. Twenty-nine percent of the students were in the 9th grade, 26% were in the 10th grade, 22% each were in 11th and 12th grades. Most students reported no lifetime use of cigarettes (79.8%) or e-cigarettes (70.6%). Fifteen percent reported lifetime but not current cigarette smoking, whereas 5% reported current cigarette use. One-fifth reported lifetime but not current e-cigarette use, and 8.6% reported current e-cigarette use. Most also indicated no past 30-day use of other combustible tobacco products such as cigars and cigarillos (92.6%), and no past 30-day use of non-combustible tobacco products including chewing tobacco, snuff, dip, or dissolvable tobacco (96.5%). The prevalence of current marijuana use, 26.1%, was higher than the prevalence for any of the tobacco use variables.

**Table 1:** Grade Level, Sex, Substance Use Among Black 9th–12th Graders, 2017 National Youth Risk Behavior Survey (*n* = 2790)

Characteristic	***n*** ***(weighted %)***
**Grade Level**	
9th	719 (29.4)
10th	717 (26.1)
11th	650 (22.5)
12th	703 (22.0)
**Sex**	
Male	1348 (49.8)
Female	1442 (50.2)
**Cigarette Use**	
Never	1558 (79.8)
Ever	326 (15.2)
Recent use	106 (5.0)
**E-Cigarette Use**	
Never	1773 (70.6)
Ever	507 (20.8)
Recent use	179 (8.6)
**Non-combustible tobacco product use (i.e., chewing tobacco, snuff, dip, or dissolvable tobacco) past 30 days**	
No	2647 (96.5)
Yes	90 (3.5)
**Combustible tobacco product use (i.e., cigars, cigarillos) past 30 days**	
No	2509 (92.6)
Yes	204 (7.4)
**Marijuana Use**	
Never	1540 (57.2)
Ever	449 (17.2)
Recent use	623 (26.6)

### Latent class analysis

The comparison of model fit indicated that a three-class solution was optimal. As shown in Table 2, the latent class distribution highlighted three distinct profiles of tobacco use behaviors among Black high school students: Class 1 (Non-Users), Class 2 (E-Cigarette Users), and Class 3 (Polytobacco Users). Non-Users comprised the largest proportion of the sample (80.9%), followed by E-cigarette users (14.3%); 4.8% were classified as Polytobacco Users. Non-users were not engaged in tobacco use. E-cigarette users had low conditional probabilities for current use of cigars and cigarillos (0.169) and chewing tobacco, snuff, dip, or dissolvable tobacco (0.039); they had higher conditional probabilities for lifetime/non-current use of cigarettes (0.480) and e-cigarettes (0.703) than the other two classes. Polytobacco users had the highest conditional probabilities for current use of cigarettes (0.738), e-cigarettes (0.771), cigars and cigarillos (0.813), and chewing tobacco, snuff, dip, or dissolvable tobacco (0.548).

**Table 2:** Latent Classes of Tobacco Use Among Black 9th–12th Graders, 2017 National Youth Risk Behavior Survey (*n* = 2782)

Latent Class Indicators	Class 1: Non-Users n (%) 2250 (80.9)	Class 2: E-Cigarette Users n (%) 399 (14.3)	Class 3: Polytobacco Users n (%) 133 (4.8)
**Cigarette use**			
Never	0.907	0.467	0.149
Ever	0.087	0.480	0.113
Recent use	0.006	0.053	*0.738*
**E-cigarette use**			
Never	0.884	0.005	0.209
Ever	0.115	*0.703*	0.020
Recent (30 day) use	0.000	0.292	*0.771*
**Cigar Use (past 30 days)**			
No	0.997	0.831	0.187
Yes	0.003	0.169	*0.813*
**Dip/chewing tobacco Use (past 30 days)**			
No	1.000	0.961	0.452
Yes	0.000	0.039	*0.548*

Table 3 presents results from the multinomial logistic regression to explore sex, grade level, and marijuana use in association with classification relative to Non-Users. Relative to females, males had four times greater odds of being classified as Polytobacco Users class versus the non-user class (aOR = 4.28, 95% CI: 1.62–11.35). Regarding grade level, ninth graders were less likely than twelfth graders to be classified as Polytobacco Users versus Non-users (aOR = 0.32, CI: 0.12–0.88). Students reporting current marijuana use (relative to never use) were statistically more likely to be classified as E-cigarette Users (aOR = 26.13, CI: 8.84–77.24) and Polytobacco Users (aOR = 24.61, CI: 6.95–87.11) than Non-users. Students reporting ever using marijuana use (relative to never using) had greater odds of being classified as E-cigarette Users than Non-Users (aOR = 12.45, CI: 4.05–38.29).

**Table 3:** Multinomial Regression of covariates associated with class membership relative to Class 1:Non-users, adjusted for sex, grade level, and marijuana use Among Black 9th–12th Graders, 2017 National Youth Risk Behavior Survey (*n* = 2595)

Covariates	E-Cigarette Users vs. Non-Users	Polytobacco Users vs. Non-Users
**Male Sex**	0.87 (0.53–1.40)	**4.28 (1.62–11.35)**
**Grade Level**		
9th	0.78 (0.38–1.59)	**0.32 (0.12–0.88)**
10th	0.75 (0.39–1.43)	0.62 (0.29–1.29)
11th	1.31 (0.56–3.04)	0.41 (0.17–1.01)
12th *(REF)*	–	–
**Marijuana Use**		
Recent (past 30 days)	**26.13 (8.84–77.24)**	**24.61 (6.95–87.11)**
Ever	**12.45 (4.05–38.29)**	1.59 (0.09–27.84)
Never *(REF)*	–	–

### Tobacco use behaviors among students in the Polytobacco user class

Table 4 shows the prevalence of each tobacco use behavior among Polytobacco Users. Estimates of the prevalence of current use were highest for cigars and cigarillos (89.2%) and cigarettes (84.5%), followed by e-cigarettes (69.6%), and chewing tobacco, snuff, dip, or dissolvable tobacco products (55.7%). Although 9.3% reported ever using cigarettes, and no students reported ever/lifetime use of e-cigarettes. Three-quarters of the students in the polytobacco class reported engaging in 2 or 3 of the four tobacco use behaviors (40.4 and 34.6%, respectively).

**Table 4:** Prevalence of tobacco use behaviors among Black adolescent Polytobacco Users (*n* = 133)

**Cigarette Use**	
Never	6.2%
Ever	9.3%
Recent (past 30 days) use	84.5%
**E-Cigarette Use**	
Never	30.4%
Ever	0.0%
Recent (past 30 day) use	69.6%
**Dip, chewing tobacco, snuff**	
No	44.3%
Yes	55.7%
**Cigars, cigarillos**	
No	10.8%
Yes	89.2%
**Number of Tobacco Use Behaviors**	
One	11.0%
Two	40.4%
Three	34.6%
Four	14.0%

Of the 133 students classified as Polytobacco Users, 67 reported seven unique combinations of tobacco use behaviors (Table 5). Specifically, more than one-fourth reported current use of all four behaviors (28.4%), 22.4% reported ever using other non-combustible tobacco products (chewing tobacco, snuff, dip, or dissolvable tobacco) and current use of the other three tobacco use behaviors (i.e., cigarettes, other combustible tobacco products (cigars and cigarillos), and e-cigarettes). Additionally, 17.8% reported use of combustible tobacco products (i.e., cigarettes and other combustible tobacco products), but not use of e-cigarettes or non-combustible tobacco products.

**Table 5:** Most prevalent unique combinations of tobacco use behaviors among Polytobacco users (*n* = 67)

E-Cigarettes	Cigarettes	Dip, chewing tobacco, snuff	Cigars, Cigarillos	n
Any past 30-day use	Any past 30-day use	Any past 30-day use	Any past 30-day use	19
Any past 30-day use	Any past 30-day use	No past 30-day use	Any past 30-day use	15
No lifetime use	Any past 30-day use	No past 30-day use	Any past 30-day use	12
Any past 30-day use	Any past 30-day use	No past 30-day use	No past 30-day use	9
No lifetime use	Lifetime use, no past 30-day use	No past 30-day use	Any past 30-day use	4
Any past 30-day use	Lifetime use, no past 30-day use	Any past 30-day use	Any past 30-day use	4
No lifetime use	Any past 30-day use	Any past 30-day use	Any past 30-day use	4

## Discussion

This study characterized patterns of polytobacco use among U.S. Black high school students. Our findings indicate that the prevalence of past 30-day use of each of the four tobacco use behaviors was < 10%. Approximately 80% of the students were best classified as Non-Users, meaning they were largely not engaged in any of the tobacco use behaviors. This is consistent with studies demonstrating large declines in tobacco use among youth [12]; however, 14% were characterized as E-cigarette Users” and approximately 5% were characterized as Polytobacco Users. The importance of this investigation is highlighted by the persistence of tobacco-related health disparities experienced by Blacks, targeted marketing of the most harmful products to Black people by the tobacco industry, and comparatively limited access to tobacco use cessation programs among Black Americans [34–36].

We found only one recent study that examined polytobacco use among Black youth [23]. The results of that study identified two classes, which were deemed “Non-Users” and “Cigarette/cigar” groups. Males were more likely to be classified in “Cigarette/cigar” group and this class had higher odds of increased nicotine dependence. Those results are similar to the findings of the current study (males reporting higher odds of multiple tobacco product use); however, a key difference is the focus on current use only and smaller sample size (*n* = 852) [23]. The current study included lifetime use, which gives us an insight into what tobacco products Black youth might be more likely to experiment with. These data could be used to design preventive interventions before youth become current or frequent tobacco product users.

Youth in the E-cigarette Users class had a 70% chance of lifetime use of e-cigarettes and nearly 50% chance of ever/lifetime smoking a cigarette. The 30% chance that youth in this class were currently using e-cigarettes is concerning. Assessments of the harm potential of e-cigarettes are very limited. The e-liquids available for these products have a wide variation in nicotine content, and the amount of nicotine in many vials could be fatal if ingested orally or transdermally [21]. The variation in the amount of nicotine and other potentially harmful ingredients contained in tobacco products as well as differences in how products are used (e.g., how often and quantity consumed) [37] increase the importance of understanding which products are being used by youth. Little is known about the true harm reduction value or addiction reduction potential of alternate products (e.g., hookah or e-cigarettes) relative to combustible cigarettes. For example, there is research that suggests one hookah session could be the equivalent of toxicant exposure of smoking 1 to 50 cigarettes [38]. Further, Freiberg et al. (2009) found that hookah produced a significantly higher carbon monoxide exposure while delivering the same amount of nicotine in a laboratory-controlled experiment compared to cigarettes [39].

Polytobacco Users had a high likelihood of current use of all four forms of tobacco/nicotine use included in the present study. Specifically, Polytobacco Users had a 54% chance of past 30-day smokeless tobacco use, 81% chance of current cigar use, and 77% chance of past 30-day electronic cigarette use. Further analysis indicates that 75% of these youth had used two or three tobacco products concurrently in the last 30 days, with cigars and cigarettes having the highest use prevalence. Our findings raise the possibility that a sizeable subgroup of Black teens may benefit from interventions targeting multiple product use, and e-cigarette and cigar use specifically. Concurrent use of tobacco products places Black polytobacco users at increases risk of poor health consequences among Black Polytobacco Users. The literature has long supported that U.S. Blacks smoke fewer cigarettes per day [1, 40], take fewer puffs per cigarette [41]—all while experiencing higher rates of tobacco-related deaths from coronary heart disease, stroke and lung cancer [1, 11].

Relative to the non-user class, males (vs. females) were more likely to be Polytobacco Users and 9th graders (vs. 12th graders) were less likely be in the Polytobacco Users class. Black males and older youth are at greater risk of being Polytobacco Users and could benefit from targeted programming. Further, current marijuana use (vs. never use) was associated with an over 20-fold increased odds of being classified as E-cigarette Users or Polytobacco Users compared to Non-Users. The present study confirms prior findings of the significant association of marijuana use with tobacco use [15, 17] and highlights the need for prevention scientists to consider comorbid intervention designs.

The present study has limitations. Data were self-reported from youth and as such substance use behaviors could have been over- or under-reported. The YRBSS survey is not all inclusive of all tobacco products (e.g., hookahs) and the data are cross-sectional, so causality cannot be determined. Notwithstanding, these findings represent a nationally representative sample of Black youth and fill an important gap in the pertinent literature.

## Conclusions

This research suggests the need to examine the unique effects of polytobacco use on Black Americans, given the excess morbidity and mortality associated with use. Tobacco use in any form is unsafe [42]; therefore, there has been increased public health concerns about use of multiple tobacco products, also referred to as polytobacco use, among U.S. adolescents [26, 27]. The harmful effects of tobacco use may be heightened when marijuana is also used [43, 44]. For example, respiratory problems were found to be more common among those who concurrently used tobacco and marijuana [17]. Future research should incorporate longitudinal assessments of the health and social consequences of polytobacco use with marijuana.

## Data Availability

Youth Risk Behavior Surveillance System (YBRSS) data are publicly available through the Centers for Disease Control and Prevention website: https://www.cdc.gov/healthyyouth/data/yrbs/index.htm
